# The digital crossroads: Media literacy and the future of youth online

**DOI:** 10.1371/journal.pdig.0000876

**Published:** 2025-06-05

**Authors:** Kate Barranco, Kendall Bryant

**Affiliations:** Red House, Georgetown University, Washington, District of Columbia, United States of America; McGill University, CANADA

Today’s youth are digitally immersed. Being a young person today *literally* goes hand-in-hand with an amalgamation of smart devices, softwares, platforms, and memes [1] that have shaped their individual and collective experiences. Actively or passively, younger generations use social media to find their way through bleak times. The real question is whether it actually moves us toward healing or paralyzes us with our own misery.

Young peoples’ paradoxical relationships with social media—which provide both agency and powerlessness, community and isolation, heightened awareness alongside desensitization—underscore the urgent need for considering media literacy and digital health citizenship as starting points for transforming their relationships to online platforms. While advocates push for protections online [2] media literacy strategies are a starting point for young users to reclaim their autonomy in the digital sphere and beyond.

Fundamentally, smartphones are tools for communication and information-seeking. Despite the good-or-bad rhetoric of discussions [3] on smartphones, these tools have the capacity for harm *and* healing, which adds to their complex effect on modern childhood. While the digi-sphere can be a joyful, fun, primed space for learning, platforms’ algorithms are designed to promote more extreme content that draws and keeps our attention, regardless of impact. [4] Whether it’s influencers promoting “how to” advertisements on uniform body types, or footage from the front lines of climate catastrophes, war, police brutality, and more, what we view online unconsciously shapes our perception of reality. As debate emerges on whether kids should have access to phones in schools, a systems thinking approach allows us to unravel the complexity of the digital paradox and how to mitigate harm while empowering youth.

## A systems view of smart devices

Systems expert Donella Meadows describes systems as “interconnected set[s] of elements … organized in a way that achieves something” [5]. David Peter Stroh extrapolates further, noting that systems thinking requires understanding systems through their “espoused” or stated purpose versus their “current” or actual purpose by analyzing the cause-and-effect relationships they facilitate [5]. For example, while tech companies adopt lofty purpose-driven missions centered on “connection*”* [6] or “empower[ment]” [7], a systems view of smartphones and social platforms shows us that their stated purpose doesn’t encapsulate the full story. Social media’s intentional, attention-capturing design [8,9–11] allows companies to excessively profit off our most raw psychological capacities, like fear, anger, and the compulsive desire for gratification. Algorithms controlling our feeds, the slot-machine-like infinite scroll, and instant gratification from the like button reinforce a sense of deep-seeded powerlessness in the adolescent psyche. Not to mention the never-ending stream of notifications: young people report receiving nearly 242 notifications per day on average [12]. As “master’s tools” [13] built for profit maximization and surveillance, users seem to tangibly lose agency over their time, behavioral patterns, and cognitive load in the name of artificial intimacy. For young people, smart devices aren’t just tools. Social media use intertwines with the process of self-discovery, presenting challenges that extend beyond the conventional tribulations of adolescence. These hurdles involve discerning the boundary between curated online personas and authentic selfhood, understanding the implications of our digital footprints, and negotiating the intricate dance between connectivity and solitude. While not all content is equal in its impact, the pressing question is: what are the psychological effects of growing up with compounded exposure inside the digital ecosystem, if the system and its users remain unchanged?

With knowledge, comes power—but also the unsettling weight of powerlessness. Social media gives users access to an awareness that has the potential to empower changemakers in new ways, but it also has the potential to incite a new crisis—a crisis of hope. Platforms and algorithms have been extensively designed to be addictive. It is time for a sustained effort to give youth the tools to safely navigate the daunting digital landscape, reclaim their attention, and empower their agency.

## Media literacy as an intervention point

Designing sustainable and humane systems while mitigating psycho-social harm requires multiple tools, techniques, and changes that operate both on the technologies themselves and their users. One strategy that can be widely integrated is media literacy, a key component of Digital Health Citizenship. According to Media Literacy Now, media literacy is “... the ability to decode media messages (including the systems in which they exist); assess the influence of those messages on thoughts, feelings, and behaviors; and create media thoughtfully and conscientiously” [14]. To us, prioritizing media literacy means giving young people the tools to both interpret the content they see online *and* have an awareness of how their technologies and platforms impact their minds. Most media literacy approaches stop at teaching users how to access multiple sources to gather information and fact-check the claims they find on the internet. But, we don’t believe that media literacy should be limited to analyzing information. These methods should be fortified with a systems approach that highlights the designs of social media platforms and their psychological impacts. For example, young people who learn the basics about how algorithms generate their feeds will have more of a holistic understanding of how to engage online. They can understand why certain posts get pushed onto their feeds and learn how to course correct when faced with harmful uploads. This expanded approach to media literacy can equip users with the resilience to navigate the impact of interacting with distressing content and potentially challenge the pattern of desensitization that is normalized online.

Without the skills to critically understand and analyze media or how those designs of social media platforms impact one’s own body and connection to (or disconnection from) others, youth find themselves trapped in vicious cycles online that foment feelings of powerlessness, desensitization, and disconnection. They are also more likely to create media that perpetuate those same vicious cycles.

On the other hand, when media literacy is integrated as an approach to social media, platforms can be used in ways that lift up agency, community, and awareness. With media literacy, developing a user profile or online presence can promote authentic self-expression. Additionally, media literacy makes greater space for community-building through the Internet, allowing young people to connect with one another around shared experiences (especially around mental health and world crises) and create material conditions for care and resensitization. Lastly, media literacy can give youth more productive ways to deal with the massive amounts of information available to them, fostering a collective sense of empowerment and compassion in the digital age.

## Conclusion

While young people, especially those on the margins, have used social media platforms in positive and productive ways to mobilize around social change, the purposefully addictive design of technologies can still trap them in a vicious cycle of maladaptive coping and the continuous “doom scroll.” We believe that strong Digital Health Citizenship requires the widespread adoption of media literacy as informed by a systems view of social media, its components, and its interconnected and desensitizing consequences. This combination will be a powerful intervention for not only generating a media-conscious citizenry but also for developing generations of resilient and connected individuals, communities, and systems as new technologies like AI emerge.

Unraveling the paradoxes, we see that social media isn’t just a problem or solution, but a topic for actively engaging in ongoing dialogue about the intergenerational impact and responsibility we owe to ourselves and future generations.

## References

[1] Merriam-Webster. Meme. In: Merriam-Webster.com Dictionary. n.d. [cited 2025 Feb 24]. Available from: https://www.merriam-webster.com/dictionary/meme

[2] Design It For Us. 2025 [cited 2025 May 2]. Available from: https://designitforus.org/

[3] Aggeler M. Are smartphones bad for us? Five world experts answer. The Guardian; 2024 [cited 2025 May 2]. Available from: https://www.theguardian.com/lifeandstyle/2024/jan/10/smartphone-screentime-good-bad-expert-advice

[4] Dizikes P. Study: on Twitter, false news travels faster than true stories. MIT News; 2018 [cited 2025 May 2]. Available from: https://news.mit.edu/2018/study-twitter-false-news-travels-faster-true-stories-0308

[5] Stroh DP. Systems thinking for social change: a practical guide to solving complex problems, avoiding unintended consequences, and achieving lasting results. White River Junction, VT: Chelsea Green Publishing; 2015. 16–17.

[6] Instagram. About Instagram. n.d. [cited 2025 May 2]. Available from: https://about.instagram.com/

[7] Apple Inc. Our values. Cupertino, CA: Apple Inc.; n.d. [cited 2025 May 2]. Available from: https://investor.apple.com/our_values/default.aspx

[8] Hari J. Stolen focus: why you can’t pay attention. New York: Bloomsbury. 2022. 118–233.

[9] Carr N. The shallows: What the internet is doing to our brains. New York: W. W. Norton; 2010.

[10] Wu T. The attention merchants: the epic scramble to get inside our heads. New York: Knopf; 2016.

[11] Ward AF. Supernormal: How the internet is changing memories and our minds. Psychol Inq. 2013;24(4):341–8.

[12] DCDX. Gen Z Screen Time Report. n.d. [cited 2025 Feb 24]. Available from: https://dcdx.co/gen-z-screen-time-report

[13] Lorde A. The master’s tools will never dismantle the master’s house. New York, NY; 1979.

[14] Media Literacy Now. What is media literacy? n.d. [cited 2025 Feb 24]. Available from: https://medialiteracynow.org/challenge/what-is-media-literacy/

